# Exploring the frequency of anemia in patients with Axial Spondyloarthritis: associations with inflammatory markers and response to Anti-Tumor Necrosis Factor (TNF)-α therapy

**DOI:** 10.7717/peerj.21178

**Published:** 2026-04-15

**Authors:** Duygu Güler, Ebru Yilmaz, Özge Pasin, Mehmet Serkan Kılıçoğlu

**Affiliations:** 1 Department of Physical Medicine and Rehabilitation, Erzurum, Turkey; 2 Department of Physical Medicine and Rehabilitation, Istanbul, Turkey; 3 Department of Biostatistics and Medical Informatics, Istanbul, Turkey

**Keywords:** Axial spondyloarthritis, Anemia of chronic disease, Anti-TNF-α therapy

## Abstract

**Background:**

Despite the well-established link between systemic inflammation and anemia in rheumatic diseases, the relationship between specific inflammatory markers and both anemia and therapeutic response in axial spondyloarthritis (axSpA) remains unclear. This study aimed to determine the frequency of anemia in patients with axSpA, assess its association with specific inflammatory markers such as the neutrophil-to-lymphocyte ratio (NLR), platelet-to-lymphocyte ratio (PLR), and systemic immune-inflammation index (SII) and evaluate the impact of anti-tumor necrosis factor (TNF)-α therapy on these parameters.

**Methods:**

This study included 102 patients with axSpA. Patient characteristics and laboratory parameters including C-reactive protein (CRP), erythrocyte sedimentation rate (ESR), hemoglobin (Hb), hematocrit (Hct) and mean corpuscular volume (MCV), and values of NLR, PLR and SII were evaluated at baseline and after 12 months of continuous anti–TNF-α therapy.

**Results:**

Anemia frequency dropped from 33.3% to 16.7% after one year of anti-TNF-α therapy. It was more common in females than males (*p* < 0.001), and anemic patients had higher ESR levels (*p* = 0.002). Hb levels significantly increased across all treatment groups (*p* = 0.001), with no significant differences between groups (*p* = 0.591). Changes in Hb were positively correlated with age, lymphocyte count, Hct, MCV, and changes in lymphocyte count (all *p* < 0.05), and negatively correlated with PLR and changes in ESR, CRP, NLR, PLR, and SII (all *p* < 0.05). Multivariate analysis showed that changes in CRP, NLR, and SII were significantly associated with improvements in Hb.

**Conclusion:**

Anemia is a common comorbidity in axSpA and appears to be closely linked to systemic inflammation. Anti-TNF-α therapy was associated with significant improvements in Hb levels and inflammatory markers over one year. These findings support the value of simple hematologic indices to monitor inflammatory burden and treatment response in axSpA.

## Introduction

Axial spondyloarthritis (axSpA) is a chronic inflammatory rheumatic disease primarily affecting the spine and sacroiliac joints, with an unclear etiology (Tanrıkulu et al., 2016; Önal & Sarıkaya, 2021; Yilmaz, 2023a; Yilmaz, 2023b). It is a progressive condition characterized by enthesis-driven inflammation, new bone formation, the development of syndesmophytes, and gradual joint ankylosis, particularly within the axial skeleton. Patients commonly experience chronic back pain, stiffness, and impaired quality of life due to persistent inflammation and structural damage (Önal & Sarıkaya, 2021; Kim & Cho, 2012). Beyond axial involvement, peripheral arthritis, enthesitis, osteoporosis and extra-articular manifestations such as uveitis, psoriasis, and inflammatory bowel disease are frequently observed (Kim & Cho, 2012). In addition to these musculoskeletal and extra-articular features, axSpA is associated with systemic consequences, among which anemia is clinically relevant (Tanrıkulu et al., 2016).

Anemia of chronic disease (ACD)—or anemia of inflammation—is a common hypoproliferative anemia that develops in the setting of persistent immun activation, including infections, malignancies, and autoimmune conditions. ACD arises from complex interactions among inflammatory cytokines, iron homeostasis, and eryhtropoiesis. Tumor necrosis factor-alpha (TNF-α)—a central pro-inflammatory mediator—contributes to ACD by impairing erythroid progenitor proliferation and restricting iron availability through reduced release of iron from the reticuloendothelial system (Tanrıkulu et al., 2016; Önal & Sarıkaya, 2021). Increasing evidence highlights the critical role of the hepcidin–iron axis in this process. Hepcidin, an acute-phase peptide hormone synthesized by the liver, regulates systemic iron metabolism by binding to the iron exporter ferroportin, inducing its internalization, and thereby reducing dietary iron absorption and iron mobilization from macrophages (Tanrıkulu et al., 2016; Önal & Sarıkaya, 2021; Niccoli et al., 2012). Elevated hepcidin levels are a hallmark of ACD, leading to iron sequestration and functional iron deficiency despite adequate or increased iron stores. Although interleukin (IL)-6 is the primary driver of hepcidin upregulation, emerging evidence suggest that TNF-α and IL-17—both central to axSpA pathogenesis—may also enhance hepcidin production, further linking chronic inflammation to disrupted iron homeostasis in this population (Tanrıkulu et al., 2016; Niccoli et al., 2012; Shu et al., 2019).

Clinically, anemia manifests with fatigue, cognitive impairment, reduced appetite, exertional dyspnea, and diminished sexual function (Kim & Cho, 2012). In rheumatoid arthritis (RA), anemia is a well-recognized extra-articular manifestation, with a prevalence of 30–70%. Similar patterns occur in axSpA, where anemia significantly contributes to reduced quality of life (Kim & Cho, 2012; Niccoli et al., 2012; Tanrıkulu et al., 2016; Önal & Sarıkaya, 2021). The hematologic impacts of inflammation have also prompted interest in complete blood count-derived inflammatory indices. Due to their accessibility and low cost, markers such as the neutrophil-lymphocyte ratio (NLR) and platelet-lymphocyte ratio (PLR) are increasingly used to reflect systemic inflammation and disease activity in rheumatic diseases (Mercan et al., 2016; Gökmen et al., 2015; Asahina et al., 2017; Tekeoğlu et al., 2016; Abd-Elazeem & Mohamed, 2018). The systemic immune-inflammation index (SII)—calculated as neutrophils × platelets/lymphocytes—has similarly emerged as a valuable tool for assessing inflammatory burden, monitoring treatment response, and even assisting in the diagnosis and prognosis of various solid organ tumors (Zhong, Huang & Chen, 2017).

Given the established influence of TNF-α in modulating both inflammation and iron metabolism, anti-TNF-α therapies may ameliorate ACD by reducing cytokine-driven hepcidin expression and restoring normal erythropoiesis. Indeed, TNF-α inhibitors not only modulate the inflammatory pathways central to axSpA but have also been shown to improve anemia in affected patients (Tanrıkulu et al., 2016; Önal & Sarıkaya, 2021; Kim & Cho, 2012; Niccoli et al., 2012; Braun et al., 2009; Bes, Yazici & Soy, 2013). However, despite accumulating evidence linking systemic inflammation, hepcidin dysregulation, and anemia in chronic rheumatic diseases, the relationships between specific inflammation-derived blood indices, anemia, and treatment response in axSpA remain insufficiently understood.

Therefore, the aim of this study is to determine the frequency of anemia in patients with axSpA, assess its association with NLR, PLR, and SII and evaluate the impact of anti-TNF-α therapy on these parameters.

## Materials and Methods

### Study design

This study was conducted as a pre–post cohort study in which patients were evaluated at baseline and again after 12 months of anti–TNF-α therapy. The study took place in the Department of Physical Medicine and Rehabilitation at Bezmialem Vakif University. Ethical approval was obtained from the Bezmialem Vakif University Ethics Committee (Trial Registration: 2023/179), and written informed consent was obtained from all participants.

### Participants and data extraction

A total of 102 patients (51 female and 51 male) diagnosed with either non-radiographic (nr) or radiographic (r) axSpA were recruited between 2023 and 2024, based on the Assessment of SpondyloArthritis International Society (ASAS) classification criteria (Deodhar, 2014). Radiographic sacroiliitis was defined according to the modified New York criteria as bilateral grade ≥2 or unilateral grade 3–4 on pelvic radiographs (Van der Linden, Valkenburg & Cats, 1984).

Inclusion criteria included: diagnosis of nr-axSpA or r-axSpA, age ≥ 18 years, and treatment with anti-TNF-α therapy with planned follow-up for at least 12 months. Exclusion criteria were: (1) history of pregnancy, alcohol abuse, malignancy, intellectual disability, severe emotional disorders, renal or liver failure, hematological diseases (*e.g.*, thalassemia), thyroid disorders, or gastrointestinal bleeding; (2) coexisting secondary rheumatic diseases such as inflammatory bowel disease (Chron’s disease, ulcerative colitis), psoriasis, psoriatic arthritis, reactive arthritis, or RA; (3) iron, vitamin B12, or folate deficiencies; (4) use of medications known to affect erythropoiesis or iron metabolism.

### Anti–TNF-α therapy

Patients received one of the following anti–TNF-α agents: etanercept, adalimumab, certolizumab pegol, golimumab, or infliximab. Standard dosing regimens were administered in accordance with clinical guidelines:

*Etanercept: 50 mg subcutaneously once weekly

*Adalimumab: 40 mg subcutaneously every other week

*Certolizumab pegol: 400 mg at weeks 0, 2, and 4, followed by 200 mg every 2 weeks

*Golimumab: 50 mg subcutaneously once monthly

*Infliximab: 5 mg/kg intravenously at weeks 0, 2, and 6, then every 6–8 weeks

To be included in the final analysis, patients were required to maintain continuous treatment for the full 12-month period. No dose escalations or prolonged treatment interruptions occurred in the study cohort.

### Data collection

Demographic and clinical variables included age, sex, weight, body mass index (BMI), smoking status, disease duration, and human leukocyte antigen (HLA)-B27 status. Laboratory parameters obtained at baseline and 12 months included:

*erythrocyte sedimentation rate (ESR), C-reactive protein (CRP),

*hemoglobin (Hb), hematocrit (Hct), mean corpuscular volume (MCV),

*neutrophil-lymphocyte ratio (NLR), platelet-lymphocyte ratio (PLR), systemic immune-inflammation index (SII).

Anemia was defined according to World Health Organization (WHO) criteria as Hb <12 mg/dL for women and <13 mg/dL for men. Anemia status was evaluated at both time points to assess changes over the treatment period. All blood samples were analyzed in the same laboratory using standardized automated hematology analyzers.

### Study design considerations and potential confounders

This study was observational and lacked a control group; therefore, causal relationships cannot be definitively established. Additionally, several potential confounders—including nonsteroidal anti-inflammatory drug (NSAID) use, disease activity indices, and iron metabolism markers such as ferritin, transferrin saturation, and hepcidin—were not recorded. These unmeasured factors may influence Hb levels and inflammatory markers and should be considered when interpreting the findings.

### Time-course analysis

Baseline and 12-month measurements were available for all participants. No intermediate follow-up time points were collected, preventing detailed time-course analysis. The pre–post comparison therefore reflects the overall longitudinal change in Hb levels and inflammatory indices following one year of continuous anti–TNF-α therapy.

### Statistical analysis

Descriptive statistics for qualitative variables are presented as numbers and percentages, while descriptive statistics for quantitative variables are provided as mean, standard deviation, median, minimum and maximum values. The normality of quantitative variables was assessed using the Kolmogorov–Smirnov test. Homogeneity of variance was evaluated with the Levene’s test. Relationships between qualitative variables were examined using Pearson’s chi-square test. The comparison of means between two independent groups was performed with the independent *t*-test (Student’s *t*-test), and the comparison of medians between two independent groups was done using the Mann–Whitney U test. The paired *t*-test was used to compare the means of two dependent groups, while the Wilcoxon signed-rank test was applied to compare the medians of two dependent groups. The Kruskall-Wallis test was used for the median comparison of more than two independent groups, with Dunn test seeving as a post-hoc test for pairwise comparisons. Pearson and Spearman correlation analysis were used to evaluate the relationships between quantitative variables. Given the large number of univariate tests performed, the False Discovery Rate (FDR) procedure was adjusted using the Benjamini–Hochberg procedure to control for multiple testing. To identify the factors affecting changes in Hb levels, variables that were significant in univariate analyses were included in a linear regression model for multivariate analysis. Also, multicollinearity was assessed using correlation analysis prior to the multivarite analysis. The final multivariable model was established by considering both statistical evidence and clinical relevance. A statistical significance level of 0.05 was considered for all calculations, and IBM SPSS Statistics for Windows, Version 26 (Armonk, NY, IBM Corp), was used for the analysis.

## Results

The demographic and clinical characteristics of the patients are presented in Table 1. All patients received an anti-TNF-α agent (nine with etanercept, 27 with adalimumab, 63 with certolizumab pegol, two with golimumab, and one with infliximab). The mean age of the study population was 42.97 ± 10.76 years. Anemia was present in 33.3% of patients (34 individuals; eight males and 26 females), and the mean Hb level was 13.07 ± 1.79 g/dL. Anemia showed no statistically significant association with smoking status or HLA-B27 positivity (*p* = 0.251 and *p* = 0.366, respectively). However, the frequency of anemia was significantly more prevalent in females than in males (*p* < 0.001, FDR-p = 0.004). Patients with anemia had significantly higher ESR levels compared to those without anemia (*p* = 0.002, FDR-p = 0.006), while RBC, Hct and MCV values were significantly lower (*p* < 0.001, FDR-p = 0.004) (Table 1). Following one year of anti-TNF-α therapy, the anemia rate was reduced by half, to 16.7% (17 individuals; three males and 14 females).

**Table 1:** The comparisons between patients with and without anemia before anti-TNF-α therapy.

Variables	Total patients	Patients with anemia	Patients without anemia	*p* value	FDR *p* value
Gender					
Female	50% (51)	25.5% (26)	24.5% (25)	<0.001	0.004
Male	50% (51)	7.8% (8)	42.2% (43)		
Age (years)	42.97 ± 10.76	45.12 ± 10.54	41.90 ± 10.79	0.155	0.368
Body mass index (BMI)	28.77 ± 5.32	30.06 ± 6.31	28.12 ± 4.67	0.261	0.495
Smoking					
Yes	28.4% (29)	6.9% (7)	21.6% (22)	0.251	0.495
No	71.6% (73)	26.5% (27)	45% (46)		
Disease duration (years)	4.89 ± 4.76	4.71 ± 4.10	4.98 ± 5.08	0.830	0.913
HLA-B27					
Positive (+)	68.6% (70)	20.6% (21)	48% (49)	0.366	0.632
Negative (-)	31.4% (32)	12.8% (13)	18.6% (19)		
CRP	13.37 ± 20.5	18.11 ± 27.95	11.01 ± 15.19	0.824	0.913
ESR	15.55 ± 13.56	22.88 ± 17.45	11.88 ± 9.29	0.002	0.006
WBC	7.96 ± 2.02	7.93 ± 1.88	7.97 ± 2.10	0.992	0.992
Neutrophil count	4.77 ± 1.75	4.72 ± 1.68	4.79 ± 1.80	0.739	0.913
Lymphocyte count	2.30 ± 0.63	2.29 ± 0.59	2.30 ± 0.65	0.629	0.853
Platelet count	270.81 ± 77.98	292.09 ± 88.32	260.18 ± 70.57	0.135	0.366
RBC	4.78 ± 0.56	4.50 ± 0.59	4.918 ± 0.480	<0.001	0.004
Hemoglobin (Hb)	13.07 ± 1.79	11.19 ± 0.96	14.01 ± 1.29	<0.001	0.004
Hematocrit (Hct)	40.34 ± 4.55	35.94 ± 3.16	42.53 ± 3.41	<0.001	0.004
MCV	84.77 ± 6.01	80.55 ± 6.99	86.88 ± 4.10	<0.001	0.004
NLR	2.240 ± 1.022	2.30 ± 1.07	2.21 ± 1.00	0.865	0.913
PLR	126.12 ± 50.82	138.66 ± 66.23	119.85 ± 40.17	0.478	0.756
SII	626.12 ± 414.05	676.90 ± 459.22	600.74 ± 390.65	0.609	0.853

**Notes.**

*p* < 0.05, significant difference.

CRP C-reactive protein ESR Erythrocyte sedimentation rate WBC White blood cell count RBC Red blood cell count MCV Mean corpuscular volume NLR Neutrophil-lymphocyte ratio PLR Platelet-lymphocyte ratio SII Systemic inflammatory immune index FDR False Discovery Rate

Correlations between Hb levels and other parameters prior to anti-TNF-α therapy are presented in Table 2. In the overall patient group, Hb levels showed positive correlations with WBC count, neutrophil count, RBC, Hct and MCV (*p* = 0.005 and FDR-p = 0.010, *p* = 0.008 and FDR-p = 0.013, *p* < 0.001 and FDR-p = 0.002, *p* < 0.001 and FDR-p = 0.002, and *p* < 0.001 and FDR-p = 0.002, respectively), and a negative correlation with ESR (*p* < 0.001, FDR-p = 0.002). Among patients with anemia, Hb levels were positively correlated with RBC and Hct (*p* = 0.001 and FDR-p = 0.005, and *p* < 0.001 and FDR-p = 0.005, respectively).

**Table 2:** The correlations between hemoglobin level and other parameters before anti-TNF-α therapy.

Variables	Hemoglobin level								
	Total patients			Patients without anemia			Patients with anemia		
	*r* value	*p* value	FDR *p* value	*r* value	*p* value	FDR *p* value	*r* value	*p* value	FDR *p* value
Disease duration (years)	0.142	0.155	0.221	0.352	0.003	0.004	0.133	0.454	0.833
ESR	−0.345	<0.001	0.002	−0.154	0.208	0.231	−0.032	0.858	0.858
WBC	0.274	0.005	0.010	0.465	<0.001	0.002	0.205	0.244	0.813
Neutrophil count	0.262	0.008	0.013	0.471	<0.001	0.002	0.099	0.577	0.833
Platelet count	−0.006	0.952	0.952	0.366	0.002	0.003	−0.097	0.585	0.833
RBC	0.663	<0.001	0.002	0.714	<0.001	0.002	0.531	0.001	0.005
Hematocrit (Hct)	0.941	<0.001	0.002	0.896	<0.001	0.002	0.871	<0.001	0.005
MCV	0.429	<0.001	0.002	0.087	0.483	0.483	0.133	0.455	0.833
NLR	0.117	0.243	0.270	0.282	0.020	0.025	0.073	0.683	0.833
SII	0.120	0.230	0279	0.385	0.001	0.002	0.057	0.750	0.833

**Notes.**

*p* < 0.05, significant difference.

ESR Erythrocyte sedimentation rate WBC White blood cell count RBC Red blood cell count MCV Mean corpuscular volume NLR Neutrophil-lymphocyte ratio SII Systemic inflammatory immune index FDR False Discovery Rate

**Figure 1:**
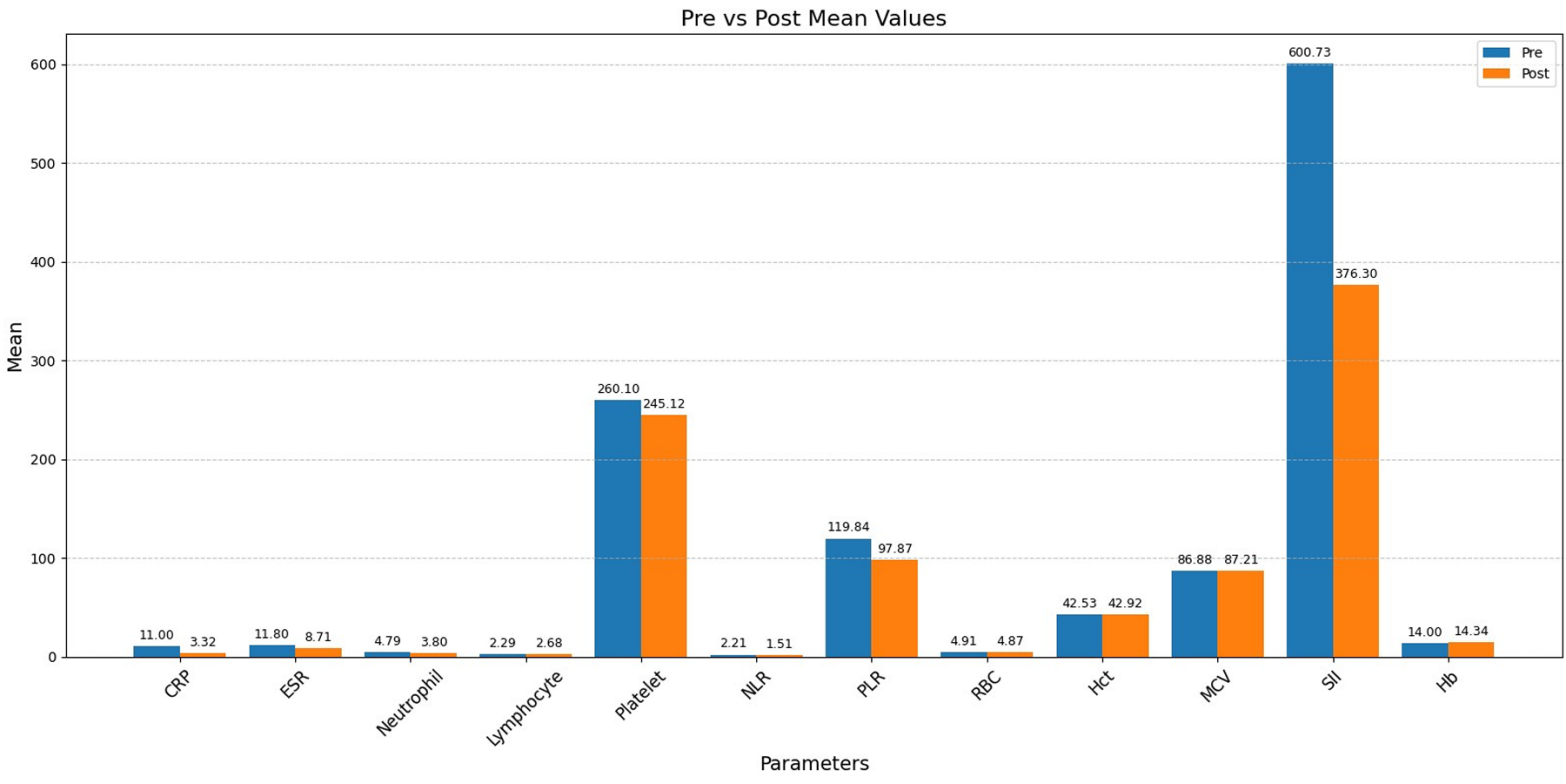
Pre- and post-treatment changes in inflammatory and hematological parameters.

Changes in hematologic and inflammatory parameters before and after 12 months of anti-TNF-α therapy are shown in Table 3 and visually summarized in Fig. 1. All parameters except platelet count and RBC count demonstrated statistically significant changes after one year of treatment. In the non-anemic subgroup, significant differences were observed in all parameters except platelet count, RBC, and Hct. In contrast, among patients with anemia, significant changes were found in all parameters except platelet count and WBC count.

**Table 3:** The changes in blood parameters before and one year after anti-TNF-α therapy.

Variables	Total patients		*p* value	FDR *p* value	Patients with anemia		*p* value	FDR *p* value	Patients without anemia		*p* value	FDR *p* value
	Before	After			Before	After			Before	After		
CRP	13.37 ± 20.49	3.21 ± 7.56	<0.001	0.001	18.10 ± 27.95	2.97 ± 4.31	0.003	0.004	11.01 ± 15.20	3.33 ± 8.77	<0.001	0.002
ESR	15.55 ± 13.56	9.28 ± 7.39	<0.001	0.001	22.88 ± 17.45	10.44 ± 7.39	<0.001	0.001	11.88 ± 9.30	8.71 ± 7.38	0.006	0.007
WBC	7.96 ± 2.02	7.42 ± 1.65	0.004	0.004	7.93 ± 1.88	7.59 ± 1.64	0.344	0.372	7.97 ± 2.10	7.34 ± 1.67	0.003	0.005
Neutrophil	4.77 ± 1.75	3.86 ± 1.25	<0.001	0.001	4.72 ± 1.68	3.97 ± 1.18	0.010	0.013	4.79 ± 1.80	3.81 ± 1.29	<0.001	0.002
Lymphocyte	2.30 ± 0.63	2.771 ± 0.697	<0.001	0.001	2.29 ± 0.59	2.94 ± 0.69	<0.001	0.001	2.30 ± 0.65	2.69 ± 0.69	<0.001	0.002
Platelet	270.81 ± 77.98	254.52 ± 55.01	0.090	0.975	292.09 ± 88.32	273.32 ± 50.06	0.561	0.561	260.18 ± 70.57	245.12 ± 55.31	0.127	0.150
RBC	4.78 ± 0.55	4.80 ± 0.49	0.481	0.481	4.50 ± 0.59	4.65 ± 0.57	0.017	0.020	4.92 ± 0.48	4.88 ± 0.44	0.289	0.289
Hb	13.07 ± 1.79	13.69 ± 1.69	<0.001	0.001	11.19 ± 0.96	12.36 ± 1.50	<0.001	0.002	14.01 ± 1.29	14.35 ± 1.37	<0.001	0.002
Hct	40.34 ± 4.55	41.52 ± 4.25	<0.001	0.001	35.94 ± 3.16	38.70 ± 3.95	<0.001	0.002	42.53 ± 3.41	42.92 ± 3.67	0.202	0.218
MCV	84.77 ± 6.01	86.23 ± 7.36	<0.001	0.001	80.55 ± 6.99	84.25 ± 6.44	<0.001	0.002	86.88 ± 4.10	87.22 ± 7.63	0.006	0.008
NLR	2.24 ± 1.02	1.46 ± 0.62	<0.001	0.001	2.30 ± 1.07	1.353 ± 0.464	<0.001	0.002	2.21 ± 1.00	1.51 ± 0.68	<0.001	0.002
PLR	126.12 ± 50.82	97.47 ± 34.32	<0.001	0.001	138.66 ± 66.23	96.68 ± 26.15	<0.001	0.002	119.84 ± 40.16	97.87 ± 37.93	<0.001	0.002
SII	626.12 ± 414.05	378.24 ± 181.60	<0.001	0.001	676.90 ± 459.22	382.12 ± 149.32	<0.001	0.002	600.74 ± 390.65	376.30 ± 196.78	<0.001	0.002

**Notes.**

*p* < 0.05, significant difference.

CRP C-reactive protein ESR Erythrocyte sedimentation rate WBC White blood cell count RBC Red blood cell count Hb Hemoglobin Htc Hematocrit MCV Mean corpuscular volume NLR Neutrophil-lymphocyte ratio PLR Platelet-lymphocyte ratio SII Systemic inflammatory immune index FDR False Discovery Rate

Hb levels increased significantly in all treatment groups following therapy (*p* = 0.001); however, no significant differences were observed between groups in the magnitude of Hb change (*p* = 0591) (Table 4). No statistically significant associations were identified between changes in Hb and gender, smoking status, or HLA-B27 positivity (*p* = 0.342, *p* = 0.514, *p* = 0.084, respectively).

**Figure 2:**
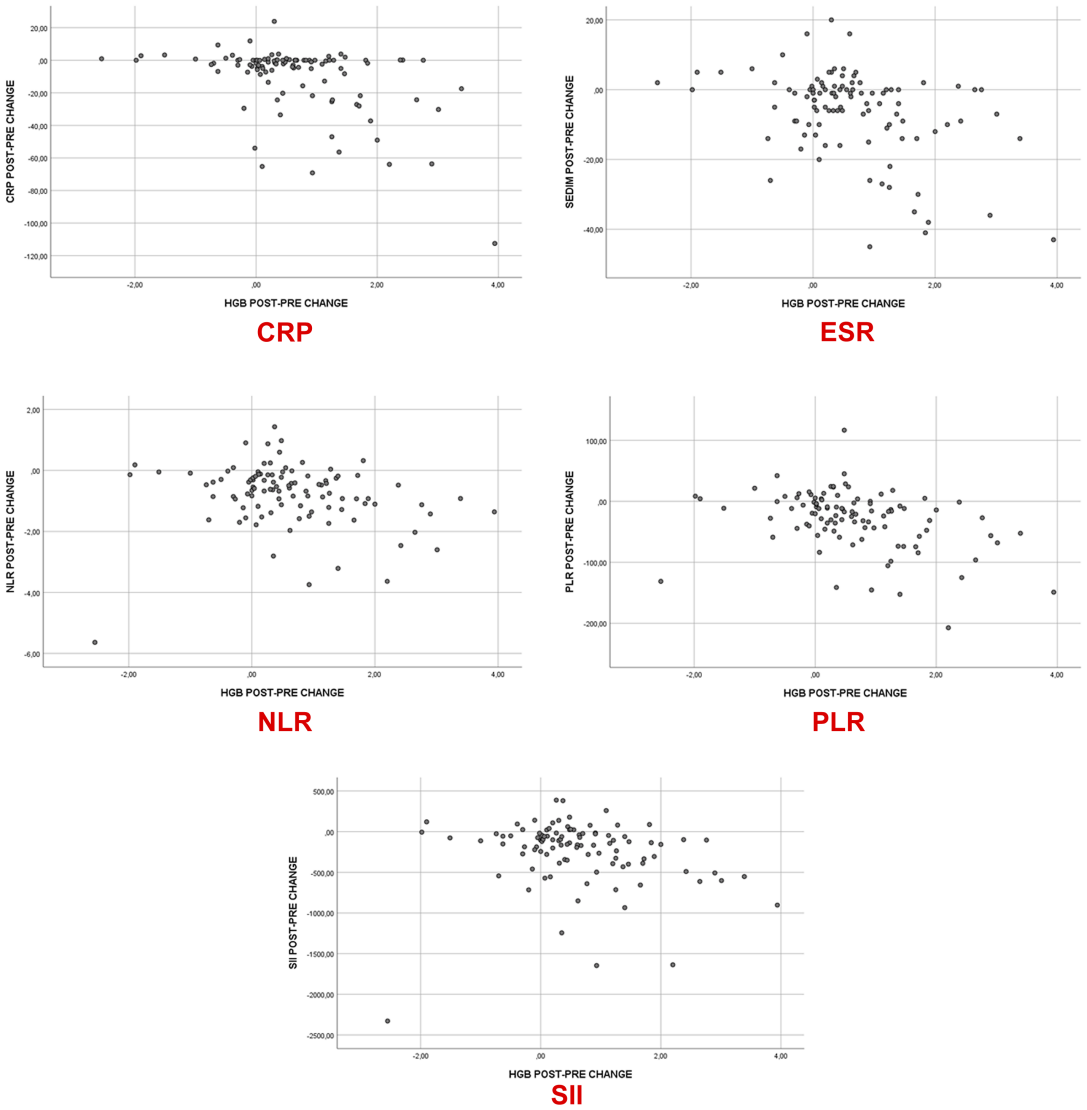
Scatter plot analysis of hemoglobin change and inflammatory marker changes.

Changes in Hb showed positive correlations with age, lymphocyte count, Hct, MCV, and change in lymphocyte count (*p* = 0.037 and FDR-p = 0.037, *p* = 0.031 and FDR-p = 0.034, *p* = 0.001 and FDR-p = 0.002, *p* = 0.004 and FDR-p = 0.005, and *p* < 0.001 and FDR-p = 0.002, respectively). Conversely, Hb change was negatively correlated with PLR and with changes in ESR, CRP, NLR, PLR, and SII (*p* = 0.010 and FDR-p = 0.012, *p* < 0.001 and FDR-p = 0.002, *p* < 0.001 and FDR-p = 0.002, *p* = 0.003 and FDR-p = 0.004, *p* < 0.001 and FDR-p = 0.002, and *p* = 0.003 and FDR-p = 0.004, respectively) (Table 5). These associations are illustrated in Fig. 2. In the multivariate linear regression analysis, changes in CRP, NLR and SII remained significantly associated with changes in Hb levels (Table 6).

## Discussion

Anemia is a common comorbidity in patients with axSpA, with reported prevalence ranging from 15% to 56.5% (Braun et al., 2009; Bes, Yazici & Soy, 2013; Kim & Cho, 2012; Niccoli et al., 2012; Önal & Sarıkaya, 2021; Tanrıkulu et al., 2016; Yilmaz & Pasin, 2025). In this study, anemia was observed in 33.3% of patients at baseline, aligning with the exisiting literature. After 12 months of anti–TNF-α therapy, this proportion decreased to 16.7%, indicating a substantial improvement. Importantly, anemia was significantly more frequent in women, consistent with broader epidemiologic patterns that may reflect sex-related physiological differences or a greater inflammatory burden among female patients. This highlights the need for gender-sensitive monitoring and further investigation. No significant associations were identified between anemia and smoking status or HLA-B27 positivity.

**Table 4:** The changes in hemoglobin levels before and one year after anti-TNF-α therapy.

Variables	Hb before treatment	Hb after treatment	*p* value	Change in Hb	*p* value
Infliximab (*n* = 1)	14.03	14.17	0.001	0.14	0.591
Etanercept (*n* = 9)	13.27 ± 2.01	13.96 ± 1.68		0.69 ± 1.76	
Adalimumab (*n* = 27)	13.86 ± 1.65	14.74 ± 1.49		0.88 ± 1.34	
Certolizumab pegol (*n* = 63)	12.65 ± 1.70	13.17 ± 1.58		0.52 ± 0.77	
Golimumab (*n* = 2)	14.01 ± 2.01	14.30 ± 1.92		0.15 ± 1.48	

**Notes.**

*p* < 0.05, significant difference.

Hb Hemoglobin

**Table 5:** The correlations among differences in hemoglobin levels and other parameters.

Variables	Change in hemoglobin		
	*r* value	*p* value	FDR *p* value
Age (years)	0.206	0.037	0.037
Lymphocyte count (before treatment)	0.214	0.031	0.034
PLR (before treatment)	−0.253	0.010	0.012
Hct (before treatment)	0.313	0.001	0.002
MCV (before treatment)	0.283	0.004	0.005
Change in CRP (before and after treatment)	−0.329	<0.001	0.002
Change in ESR (before and after treatment)	−0.332	<0.001	0.002
Change in lymphocyte count (before and after treatment)	0.349	<0.001	0.002
Change in NLR (before and after treatment)	−0.288	0.003	0.004
Change in PLR (before and after treatment)	−0.395	<0.001	0.002
Change in SII (before and after treatment)	−0.293	0.003	0.004

**Notes.**

*p* < 0.05, significant difference.

PLR Platelet-lymphocyte ratio Hct Hematocrit MCV Mean corpuscular volume CRP C-reactive protein ESR Erythrocyte sedimentation rate NLR Neutrophil-lymphocyte ratio SII Systemic inflammatory immune index FDR False Discovery Rate

The pathophysiology of anemia in axSpA is principally attributable to chronic inflammation. Persistent elevations in pro-inflammatory cytokines—including TNF-α, IL-1β, and IL-6—disrupt erythropoiesis, impair iron mobilization, and stimulate hepatic hepcidin production (Bes, Yazici & Soy, 2013; Madu & Ughasoro, 2017; Yilmaz, 2023a; Yilmaz, 2023b). Hepcidin, the principal regulator of iron homeostasis, promotes iron sequestration in macrophages and reduces intestinal absorption, resulting in functional iron deficiency even when total iron stores are preserved. This TNF–hepcidin–iron axis represents a key biological mechanism underlying ACD and may partly explain the rise in Hb following anti–TNF-α therapy. Although NSAID-induced gastrointestinal bleeding may contribute to anemia in axSpA (Tanrıkulu et al., 2016; Yilmaz & Pasin, 2025), our inability to assess NSAID use limits interpretation. In this cohort, patients with anemia exhibited higher ESR values, supporting the likelihood of inflammation-driven ACD. Correlations between Hb, ESR, RBC, Hct, and MCV further reflect laboratory patterns typical of inflammation-mediated anemia. However, the absence of disease activity scores (*e.g.*, ASDAS (Ankylosing Spondylitis Disease Activity Score), BASDAI (Bath Ankylosing Spondylitis Disease Activity Index)) and iron-related parameters (ferritin, transferrin saturation, hepcidin, serum iron, vitamin B12, folate) restricts the ability to distinguish pure ACD from mixed etiologies.

Emerging inflammatory indices such as NLR, PLR, and SII have gained prominence as accessible markers of systemic inflammation. Prior research shows that SII correlates with disease activity in axSpA (Wu, Yan & Chai, 2021). Our findings support these pattern: NLR, PLR, and SII decreased significantly after one year of anti–TNF-α therapy, and changes in these indices were associated with Hb improvement. Although baseline values did not differ significantly between anemic and non-anemic patients, their longitudinal reductions appear to track improvements in inflammatory status. Notably, reductions in CRP, NLR, and SII were independently associated with increases in Hb, reinforcing their potential value as adjunct markers for monitoring inflammatory burden and treatment response.

Anti–TNF-α agents improve clinical manifestations and reduce systemic inflammation in axSpA, and previous studies have reported hematologic benefits, including increases in Hb and reductions in anemia prevalence (Braun et al., 2009; Kim & Cho, 2012; Niccoli et al., 2012). By blocking TNF-α, these therapies may indirectly reduce hepcidin expression, enhance iron availability, and thereby promote erythropoiesis (Shu et al., 2019). Despite heterogeneous treatment distribution, our study demonstrated consistent Hb improvement across all anti–TNF-α agents, suggesting a class effect. However, without measurements of iron parameters and hepcidin, we cannot confirm the mechanistic pathway; the findings indicate association rather than direct causation.

**Table 6:** The factors affecting changes in hemoglobin levels.

Variables	β	*p* value	β (%95 CI)
Sex	0.042	0.712	(−0.182)–(0.265)
Age (years)	0.004	0.465	(−0.007)–(0.015)
Change in CRP (before and after treatment)	−0.008	0.039	(−0.016)–(0.000)
Change in ESR (before and after treatment)	0.006	0.337	(−0.006)–(0.017)
Change in lymphocyte count (before and after treatment)	−0.035	0.783	(−0.288)–(0.218)
Change in NLR (before and after treatment)	−0.327	0.048	(−0.651)–(−0.003)
Change in PLR (before and after treatment)	−0.004	0.077	(−0.009)–(0.000)
Change in SII (before and after treatment)	0.001	0.020	(0.000)–(0.002)
Change in RBC (before and after treatment)	0.402	0.087	(−0.059)–(0.862)
Change in Hct (before and after treatment)	0.247	<0.001	(0.193)–(0.300)
Change in MCV (before and after treatment)	0.010	0.224	(−0.006)–(0.027)

**Notes.**

*p* < 0.05, significant difference.

CRP C-reactive protein ESR Erythrocyte sedimentation rate NLR Neutrophil-lymphocyte ratio PLR Platelet-lymphocyte ratio SII Systemic inflammatory immune index RBC Red blood cell count Hct Hematocrit MCV Mean corpuscular volume CI Confidence interval

The hematologic response to treatment was not influenced by gender, smoking status, or HLA-B27. Instead, improvements in Hb were closely linked to reductions in systemic inflammation, as evidenced by weak to moderate correlations with decreases in CRP, NLR, and SII. These results align with previous studies identifying inflammatory markers—particularly CRP and ESR—as key predictors of Hb recovery (Bes, Yazici & Soy, 2013; Furst et al., 2013; Kim & Cho, 2012; Önal & Sarıkaya, 2021; Tanrıkulu et al., 2016). The inclusion of multiple inflammatory indices (NLR, PLR, SII) and several anti–TNF-α agents strengthens the robustness of these observations. Baseline anemia prevalence aligned with earlier reports, and the significant increases in Hb alongside reductions in ESR, CRP, NLR, PLR, and SII after one year of therapy indicate that hematologic improvements primarily reflect reductions in inflammatory burden. Monitoring these indices may facilitate more individualized and comprehensive treatment assessment.

Additionally, increases in lymphocyte counts after therapy may indicate reversal of inflammation-related lymphocyte suppression, consistent with prior evidence that anti–TNF-α agents contribute to restoration of immune homeostasis (Monaco et al., 2015), suggesting broader immunomodulatory effects. Positive correlations between Hb improvement and age, lymphocyte count, Hct, and MCV, together with inverse correlations involving PLR and inflammatory marker changes, highlight the complex interplay between inflammation, iron metabolism, and erythropoiesis.

Taken together, the findings support the view that anti–TNF-α therapy improves anemia in axSpA primarily by reducing systemic inflammation and its downstream effects on the hepcidin–iron axis. Nevertheless, the absence of disease activity measures, iron parameters, NSAID data, and treatment adherence indicators limits the strength of mechanistic claims. Future studies incorporating these variables are needed to clarify the causal pathways and to better differentiate direct drug effects from generalized reductions in inflammatory activity.

### Limitations

This study has several limitations that should be considered when interpreting the findings. First, it was a single-center observational cohort study without a control group, which limits the ability to draw causal inferences. The absence of an untreated or alternative-therapy comparison arm, along with the lack of a formal power or sample size calculation, further restricts both the generalizability and statistical robustness of the results.

Second, standard disease activity indices such as ASDAS and BASDAI were not collected, limiting the evaluation of the relationship between Hb improvement and clinical disease control. Similarly, iron metabolism parameters (*e.g.*, ferritin, transferrin saturation, serum iron, hepcidin), as well as vitamin B12 and folate levels, were not assessed, restricting the ability to distinguish true anemia of inflammation from other etiologies or mixed patterns.

Third, NSAID use, which may either suppress or confound inflammatory markers, was not systematically recorded. This, in combination with missing iron parameters and disease activity scores, introduces potential confounding and makes it difficult to determine whether the observed Hb improvements reflect direct pharmacologic effects of TNF-α inhibition or the broader consequences of reduced systemic inflammation.

Finally, the relatively small subgroups for some biologic agents may limit between-drug comparisons. Despite these limitations, the results provide valuable insight into the hematologic effects of anti–TNF-α therapy in clinical practice. Nonetheless, the findings should be interpreted as associative rather than causal. Moreover, the observed Hb increases among non-anemic patients suggest that subclinical suppression of erythropoiesis may also be reversible with effective treatment.

## Conclusion

Anemia is a frequent and clinically relevant extra-articular manifestation in patients with axSpA. In this cohort, Hb levels increased and anemia prevalence decreased after 12 months of continuous anti–TNF-α therapy. These hematologic improvements were accompanied by reductions in inflammatory indices such as NLR, PLR, and SII, suggesting a relationship between systemic inflammation and anemia in axSpA. Nonetheless, given the absence of disease activity scores, iron metabolism markers, and a control group, the findings should be interpreted as associative rather than causal.

Despite these limitations, the results highlight the value of routinely assessing hematologic parameters and inflammation-related blood indices in the clinical management of axSpA. Future studies—particularly controlled designs incorporating detailed evaluations of disease activity, iron homeostasis, treatment adherence, and longitudinal time-course analyses—are needed to clarify the mechanisms through which biologic therapy influences anemia and to better define its long-term clinical implications.

##  Supplemental Information

10.7717/peerj.21178/supp-1 Supplemental Information 1 Axial spondyloarthritis raw data Each column contains the parameters examined.

10.7717/peerj.21178/supp-2 Supplemental Information 2 STROBE checklist

## Additional Information and Declarations

### Data Availability

The following information was supplied regarding data availability: The raw data is available in the Supplemental File.

### Clinical Trial Registration

The following information was supplied regarding Clinical Trial registration: None
